# Parallelogram flap versus homodigital island flap in the treatment of fingertip defects with bone exposure: a prospective controlled study

**DOI:** 10.1186/s13018-022-03214-1

**Published:** 2022-06-21

**Authors:** Yingkai Zhang, Yao Wang, Xianwei He, Jiaqi Zhou, Guoping Cai, Rongbo Wu

**Affiliations:** 1 Department of Orthopaedic Surgery, Jinshan Hospital of Fudan University, Longhang Road 1508, Shanghai, 201508 People’s Republic of China; 2 Department of Orthopaedic Surgery, Zhongshan Hospital Fudan University, Shanghai, 200032 People’s Republic of China

**Keywords:** Fingertip, Local Flaps, Defect, Coverage, Trauma, Bone exposure

## Abstract

**Purpose:**

A modified local transposition flap (we call it “parallelogram flap”) surgery was performed for fingertip injuries. This study aimed to compare the clinical effects of parallelogram flap and homodigital island flaps in fingertip reconstruction.

**Methods:**

The study collected patients who underwent parallelogram transposition flaps and homodigital island flaps to repair fingertip defects from 2019 to 2021. 150 cases (150 fingers) were included in our study. All operations were performed by one surgical team. Record the operation time, two-point discrimination (2PD), Total Active Movement (TAM) and the MHQ (Michigan Hand Questionnaire) of the injured fingers to evaluate the therapeutic effect.

**Results:**

All parallelogram (Group A) and homodigital island flap (Group B) had survived postoperatively. The operative duration of Group A (31.2 ± 3.3 min) is shorter than Group B (97.8 ± 6.1 min) (*P* < 0.05). At the 6-month follow-up, there was no difference with the two-point discrimination (2PD) of the palmar part of the flaps and the Total Active Movement (TAM) of injured figures in Group A and Group B. The MHQ summary scores in Group A (94.29 ± 3.14) were much higher than in Group B (91.73 ± 3.41) (*P* < 0.05). Evaluation of the MHQ subscale performance showed that the overall hand function, activities of daily living, work performance and pain score had no differences(*P* > 0.05), but aesthetics (92.15 ± 7.16) and satisfaction (92.45 ± 5.61) score in Group A was higher than aesthetics (86.56 ± 5.60) and satisfaction (86.72 ± 8.21) score in Group B (*P* < 0.05 for both).

**Conclusions:**

The reconstruction using parallelogram flaps is a easier and more versatile treatment with better functions, less morbidity and better aesthetics. This method is a better choice for reconstruction of fingertip injury.

## Introduction

**Fig. 1:**
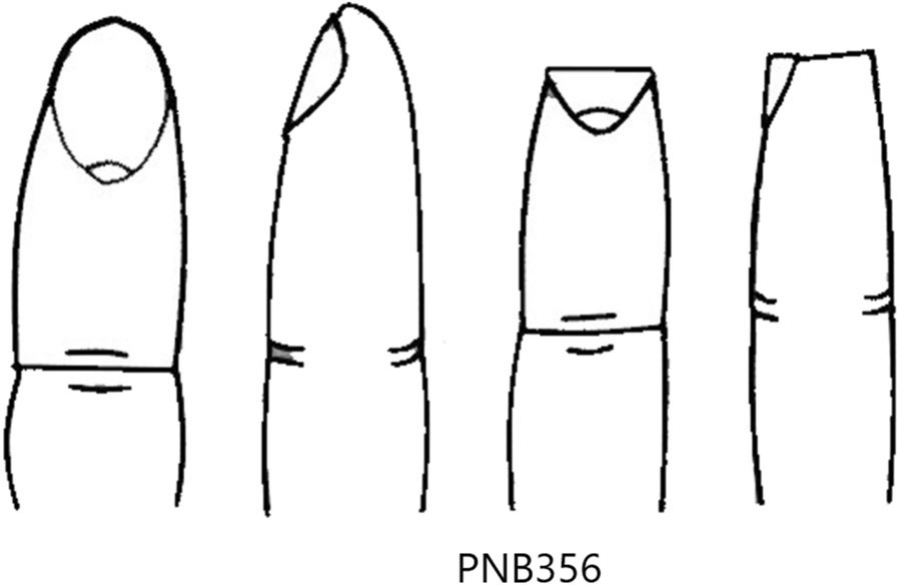
PNB356 finger amputation injuries

Finger injury is common in our daily life [1]. However, severe injuries may result in skin and soft tissue defects with the exposure of bone, joint, tendon, blood vessels and nerve, leading to disfigurement and impairment of finger function. Several approaches to repairing injured fingers are being practiced [2]. It is generally believed that amputation with sutured closure of the wound may be the most effective treatment, but patients are usually discontented due to the deficiencies of appearance and function [3]. The application of an abdominal flap allows possible rescue of injured fingers [4, 5]. However, the abdominal flap belongs to the distal flap and has several shortcomings, such as requiring multi-stage surgeries, poor wear resistance, swollen appearance, poor sense of touch, and requiring hand attachment to another part of the body for up to 3 weeks [6, 7]. While local flaps, such as the V–Y flaps have the advantages of having similar texture and sensation to the defect area, their applicability is limited when the defect area is large that the wound cannot be covered [8, 9]. To overcome these limitations, homodigital island flaps have been reported, which include a neurovascular bundle, and immediate sensory recovery is expected [10]. In this study, we aim to provide a easier and more versatile surgical technique to treat fingertip defects. Moreover, the aesthetic and function of fingers were preserved. We compare parallelogram transposition flaps and homodigital island flaps in the treatment of PNB356 finger amputation injuries [11] (Fig. 1). (Transverse amputation with the loss of distal pulp, nails, and bone was defined as PNB356).

## Methodology

### Patients

Patients undergoing parallelogram transposition flaps and the homodigital island flaps to repair fingertip defects from 2019 to 2021 were included in our prospective analyses. The study was submitted to the Ethics Committee. Patients deemed suitable for this procedure would satisfy the following inclusion criteria: (1) Single fingertip injury of one hand; (2) Transverse amputation with the loss of distal pulp, nails, and bone; (3) The injured finger had not been longer than 8 h; (4) 6 mm < Advancement required < 10 mm; (5) PNB356 finger amputation injuries (6) The patient agreed to participate at the 6-month follow-up.

Researchers coded patients in the order of admission and used SSPS20.0 software to randomly group. 150 fingers of 150 cases were treated by two types of surgery. All operations were performed by two surgical teams which had many years of clinical experience.

### Operative method

#### Wound treatment

All operations were performed by one surgical team. Firstly, the patient was given nerve block anesthesia at the root of the injured finger. A gauze was then placed at the root of the finger and tightened with a rubber band to minimize bleeding. Thorough debridement and hemostasis were performed to the wounded finger. With a partial defect of the phalange, the remnant of the phalange was repaired and the bone structure was polished. The exposed nerve stump was incised with a sharp knife so that the severed end would retract naturally into the normal soft tissue.

#### Harvesting of skin flap

##### Group A

**Fig. 2:**
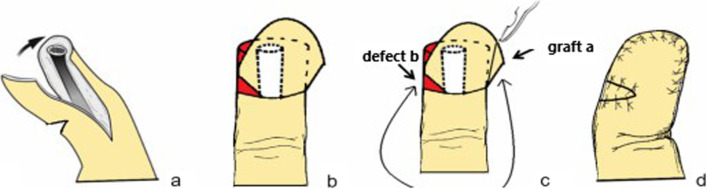
Surgical steps of the parallelogram flap: **a** A longitudinal incision was made along the bone surface on both sides of the fingertip and the incised position should not exceed the transverse striation of the distal interphalangeal joint. **b** A transverse incision was made on the side with more remaining skin to provide sufficient angle for flap turnover. **c** A piece of skin graft A was left on the opposite side and to fill the defect B. **d** The parallelogram flap reconstruction and skin grafts are completed

**Fig. 3:**
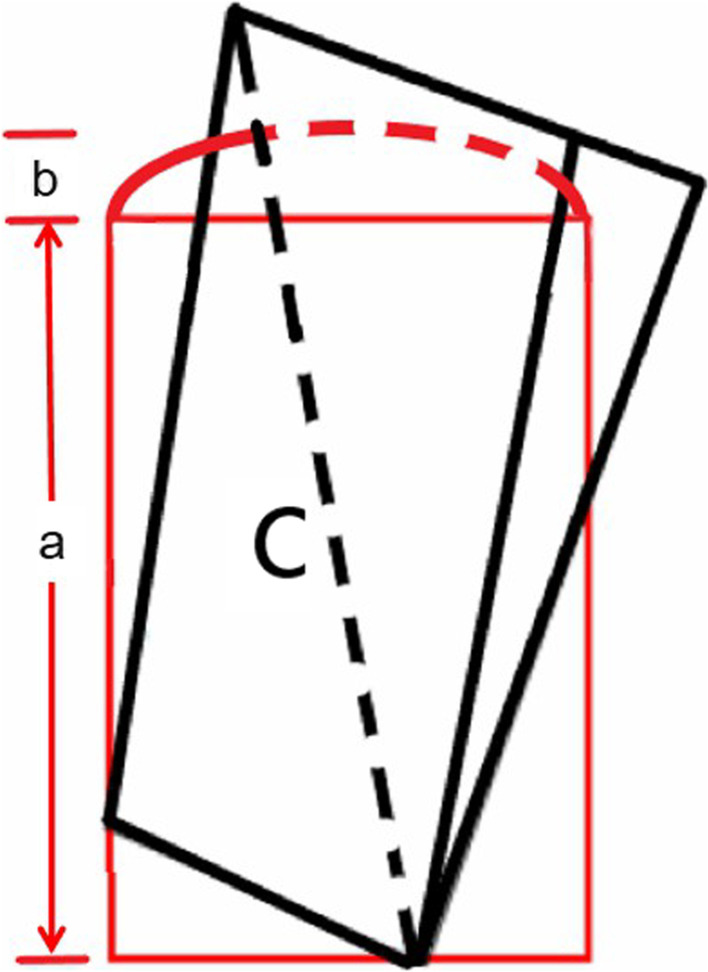
Schematic drawing of the parallelogram flap: The red represents the injured finger, and the black represents the parallelogram flap. The longest hypotenuse c should be longer than the longitudinal length a + the width of defect b

**Fig. 4:**
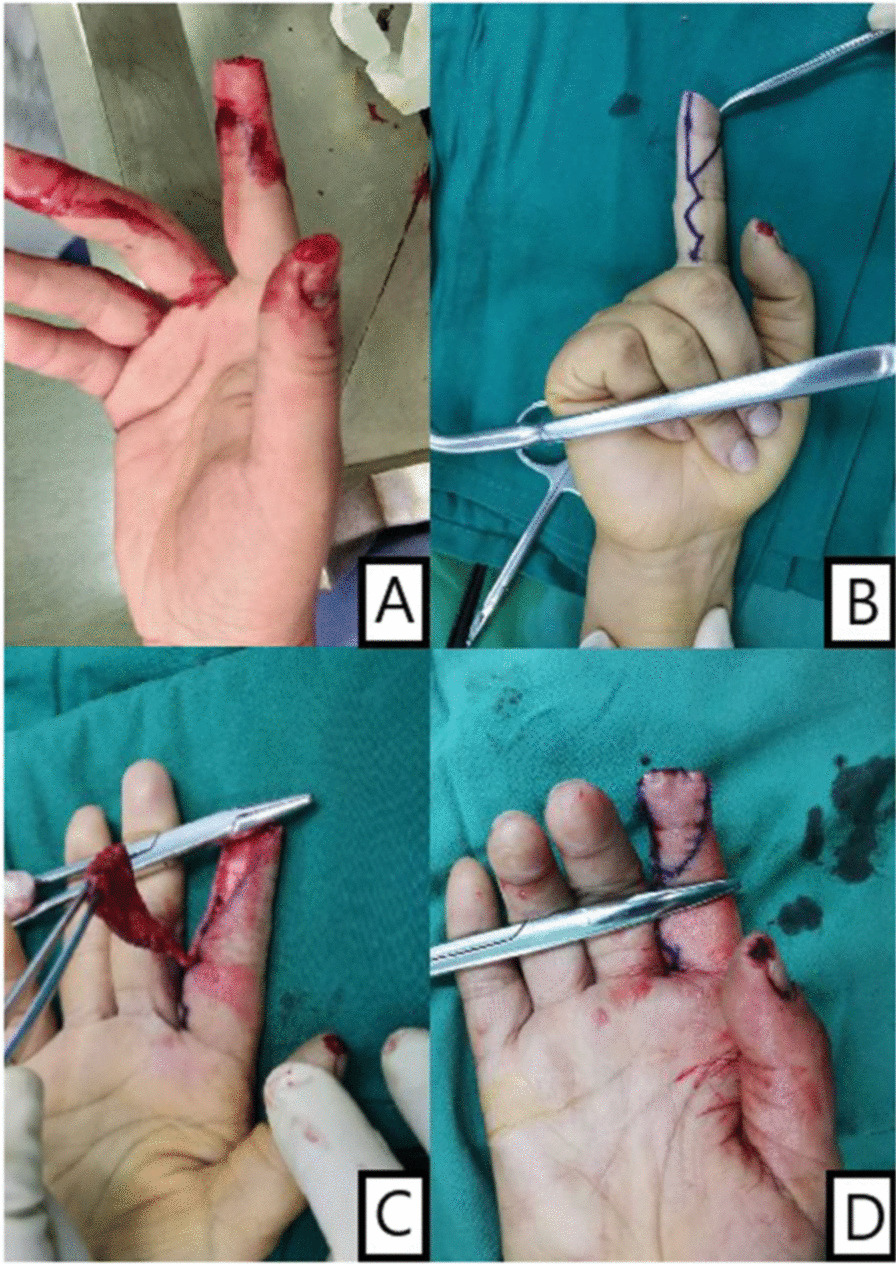
Intraoperative performance: Patients treated by homodigital island flaps

**Fig. 5:**
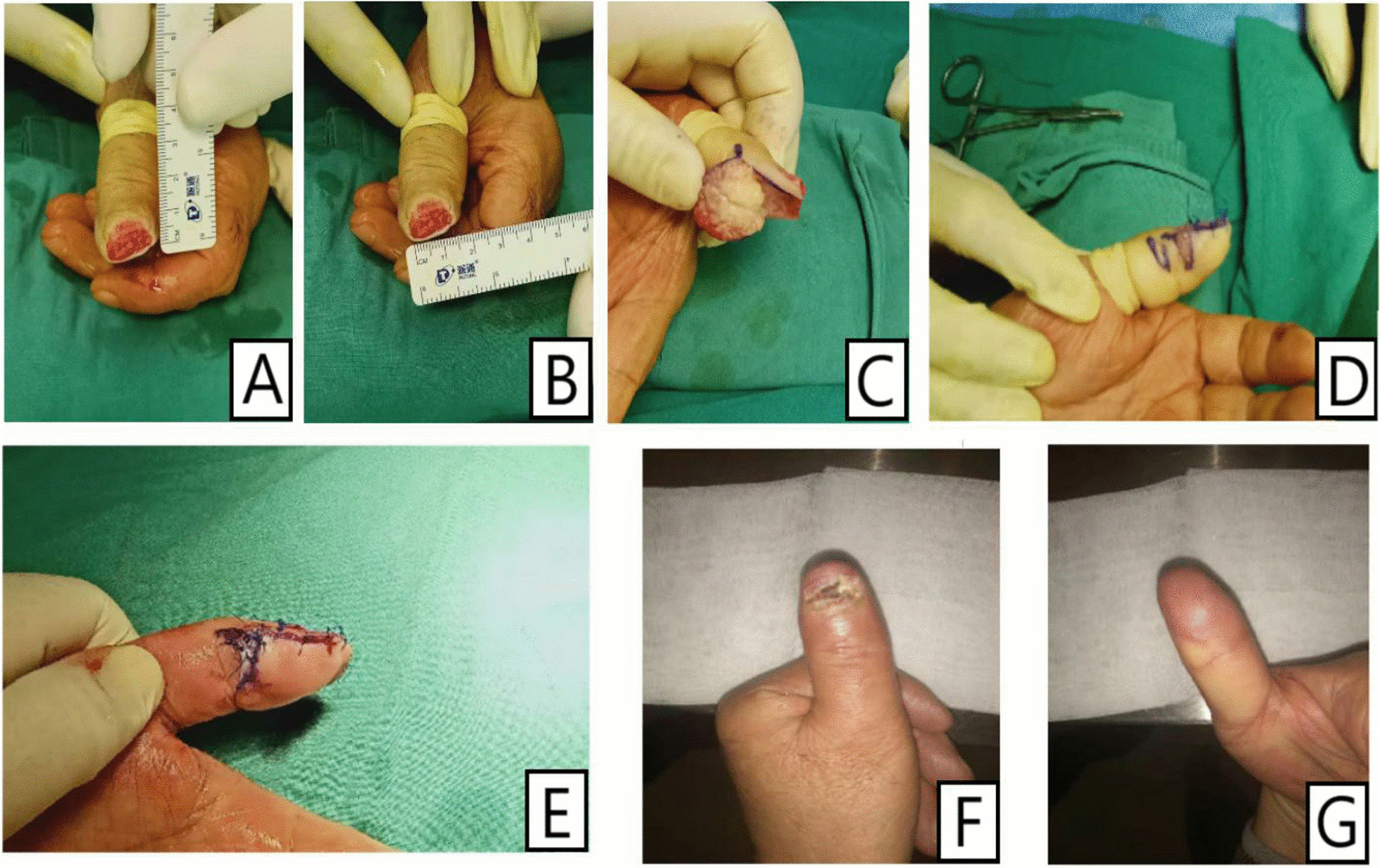
Intraoperative performance: Patients treated by parallelogram flaps **A**, **B** Postoperative performance **C**, **D**, **E** The procedure of surgery **F**, **G** four month after surgery

**Fig. 6:**
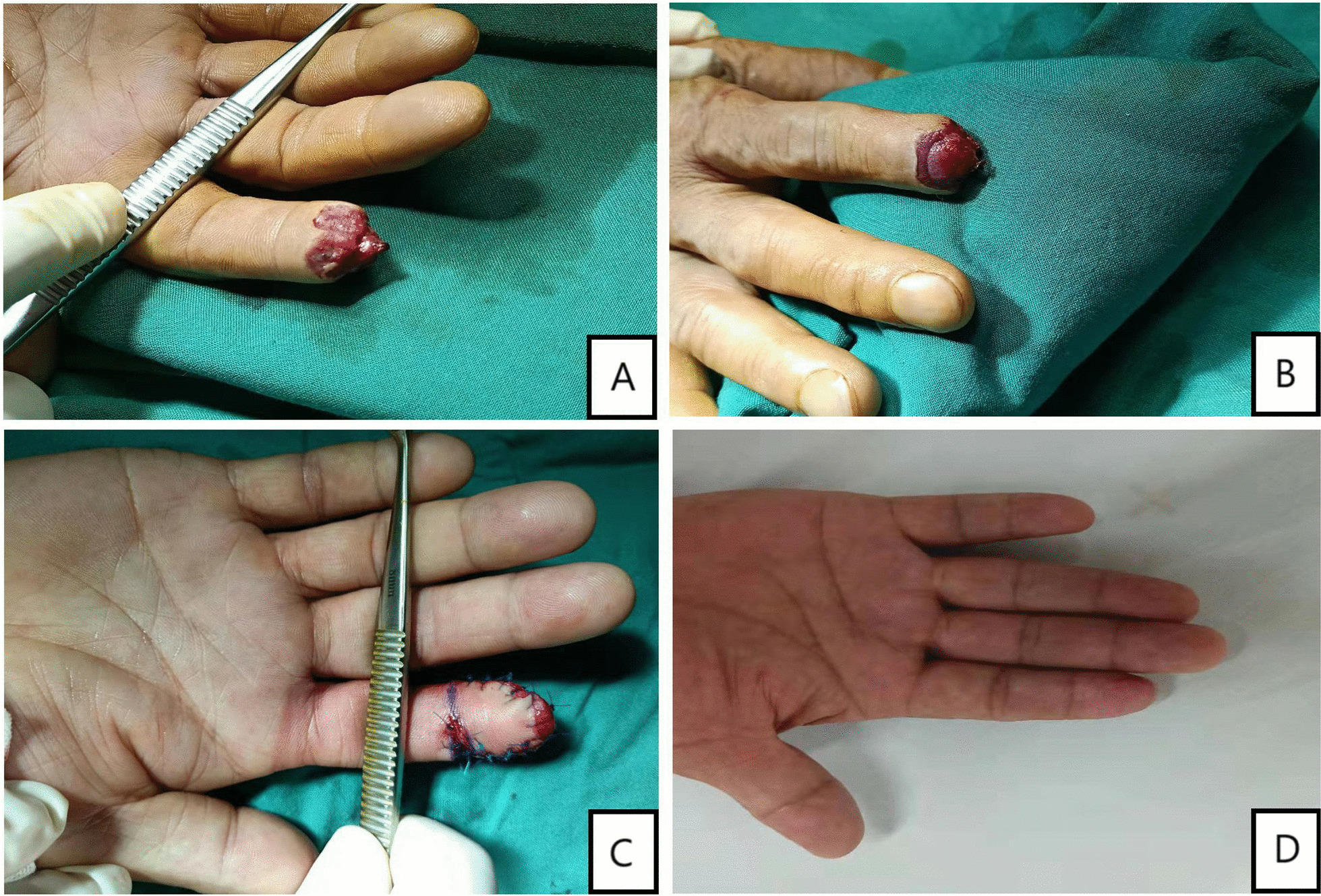
Intraoperative performance: Patients with bone exposure treated by parallelogram transposition flaps. **A**, **B** Postoperative performance **C** The procedure of surgery **D** four month after surgery

According to the size of the defect, the flap was designed on the side with more residual skin (Fig. 2). A longitudinal incision was made along the bone surface on both sides of the fingertip and the incised position should not exceed the transverse striation of the distal interphalangeal joint. Then, the skin and subcutaneous tissue were incised along the edge of the skin, and the skin flap was dissected sharply within the subcutaneous fascia, avoiding injury to the proper digital artery and nerve. A transverse incision was made on the side with more remaining skin to provide sufficient angle for flap turnover. Once freed, the designed flap was flipped over. Given its shape resembling a parallelogram, we named the flap a parallelogram flap. The longest hypotenuse c should be longer than the longitudinal length a + the width of defect b (Fig. 3), which was sufficient to cover the defective area (Fig. 4). After the flap was flipped over, a piece of skin graft A was left on the opposite side. The constructed skin graft A could be used to repair the transferred skin defect B. (Figs. 5, 6).

##### Group B

The operation was performed under local finger anesthesia. The incision was first made at the midaxis of the finger, and then the neurovascular pedicle proximal end of the flap was exposed and isolated. A finger pulp oblique incision was made to harvest the flap. Afterward, the flap was raised above the superficial flexor tendon of the finger from the distal defect area to the proximal interphalangeal. When the donor flap was raised, sufficient subcutaneous fat was incorporated to ensure maximal cosmetic value. The flap was pulled to the distal defect area in the straight position of the finger, ensuring no tension to the neurovascular bundle in the flap. Finally, the donor area was sutured directly (Fig. 4).

### Postoperative management

Postoperatively, antibiotics were given intravenously to reduce the risk of infection, in addition to lamp baking heat preservation and other symptomatic treatment. Moreover, patients received regular dressing changes and were advised to bed rest, elevate the affected limb, stop smoking, keep warm, and regularly observe the perfusion of the skin flap.

### Follow-up

At 6-month follow-up, the Total Active Movement (TAM) and two-point discrimination (2PD) were collected, and the subjective satisfaction of the patients regarding clinical efficacy was evaluated based on the MHQ (Michigan Hand Questionnaire).

1. The Total Active Movement (TAM) of the injured fingers was measured using a standard hand goniometer. The system sums the degrees of active flexion at the interphalangeal joints and metacarpophalangeal joint and subtracts the degrees of the extension deficits (100% for excellent; > 75% for good; > 50% for fair; < 50% for poor).
2. The sensibility of the palmar part of the flaps was measured using static two-point discrimination (2PD). The modfied American Society for Surgery of the Hand guidelines were used to classify the 2PD (< 6 mm for excellent; 6–10 mm for good; 11–15 mm for fair; > 15 mm for poor).
3. The MHQ (Michigan Hand Questionnaire) was used to subjectively evaluate outcomes of the repaired hands. The MHQ includes 6 subscales (overall hand function, activities of daily living, pain, work performance, aesthetics, and satisfaction).

### Statistical analysis

Data analysis was performed using the SPSS 20.0 statistical software. The Kolmogorov–Smirnov test was used to identify the normality, and all data conformed to the normal distribution. Measured data were expressed as *x* ± *s* and the independent sample *t*-test was used to compare two groups and in groups. The count data were compared by *x*^2^ test between groups. *P* values < 0.05 were considered statistically significant.

## Results

The characteristics of the study samples are detailed in Table 1. All the flaps and the skin grafts survived completely in the two groups. Patients in two groups did not differ with respect to age, gender, the cause of injury, the finger type, the interval between injury and surgery and the duration of surgery (*P* > 0.05 for each). The operative duration of Group A (31.2 ± 3.3 min) is shorter than Group B (97.8 ± 6.1 min) (*P* < 0.05) (Table 1). Accordingly, the patients’ baseline assessment indicated that the two groups were functionally similar, and the selection bias appears to have been limited.

**Table 1:** Characteristics of the sample

Characteristics of the sample	Group A	Group B	*P* value
Age (year)	43.0 SD (11.8) (range 18–66)	45.2 SD (12.1) (range 21–65)	0.258
Gender (*n*)			0.758
Male	58	63	
Female	17	12	
Cause of injury (*n*)			0.684
Twisting	26	21	
Crushing	22	25	
Cutting	27	29	
Finger type (*n*)			
Thumb	15	14	
Index fingers	17	19	
Middle fingers	31	29	
Ring fingers	8	10	
Little fingers	4	3	
Interval between injury and operation (h)	5.78 h (range 4.7–8.4 h)	5.12 h (range 4.6–8.2 h)	0.635
Operation duration (min)	31.2 ± 3.3	97.8 ± 6.1	0.018*

^*^p < 0.05 versus Group B using *t*-test

At last 6-month follow-up, there was no difference with the 2PD of the palmar part of the flaps (Table 2) and the TAM of injured figures in Group A and Group B (Table 3). The MHQ summary scores in Group A (94.29 ± 3.14) were much higher than in Group B (91.73 ± 3.41) (*P* < 0.05). Evaluation of the MHQ subscale performance showed that the overall hand function, activities of daily living, work performance and pain score had no differences (*P* > 0.05), but aesthetics (92.15 ± 7.16) and satisfaction (92.45 ± 5.61) score in Group A was higher than aesthetics (86.56 ± 5.60) and satisfaction (86.72 ± 8.21) score in Group B (*P* < 0.05 for both) (Table 4).

**Table 2:** 2PD of the palmar part of the flap

value	Group A	Group B
Excellent	13	10
Good	47	42
Fair	15	22
Poor	0	1

**Table 3:** TAM of the injured finger (*n*)

value	Group A	Group B
Excellent	59	63
Good	16	12
Fair	0	0
Poor	0	0

**Table 4:** Michigan hand outcomes questionnaire (MHQ)

Domain	Group A	Group B	*P* value
Overall hand function	93.71 (SD 3.51)	92.97 (SD 4.73)	*P* = 0.127
Activities of daily living	95.22 (SD 2.23)	94.38 (SD 3.35)	*P* = 0.09
Work performance	94.23 (SD 3.21)	94.38 (SD 3.65)	*P* = 0.374
Pain	4.34 (SD 4.01)	4.63 (SD 4.71)	*P* = 0.31
Aesthetics	92.15 (SD 7.16)	86.56 (SD 5.60)	*P* = 0.035*
Satisfaction	92.45 (SD 5.61)	86.72 (SD 8.21)	*P* = 0.027*
Summary scores	94.29 (SD 3.14)	91.73 (SD 3.41)	*P* = 0.045*

^*^p < 0.05 versus Group B using *t*-test

## Discussion

Fingertip injury represents the most common injury of the hand [12], which is defined as a distal injury of the flexor digital tendon and extensor tendon insertion [13]. In the management of a fingertip injury, although it is essential to maintain the length, preserve the nail and the appearance, the main goal of treatment is to ensure the durability of the fingertip and painless at the skin. Therefore, the treatment must be individualized based on several patient-related factors and unique trauma characteristics [14].

For those injured fingers with bone exposure and local soft tissue defects, stump revision (i.e., phalangeal shortening and direct suture) is the simplest and fastest way to recovery, which can be performed under local anesthesia in the emergency room [2]. However, this operation shortens the phalange and adversely affects the appearance and function of the affected finger. With the advancement of medical technology, stump revision is no longer a common approach to manage tissue defects [3]. Compared with stump revision, given that our method demonstrated a similar length of operative time and difficulty while retaining the length and function of the affected finger.

At present, the “V–Y” advancement flap [15] is widely performed in the management of fingertip injuries. “V–Y” flap is best used for transverse or anticlinal fingertip amputation and is suitable for injury to any finger. The contraindications of applying this flap include oblique metacarpal fingertip amputation and extensive palmar soft tissue defects. The maximum advancement distance of the skin flap is limited to 3–4 mm [16] The parallelogram transfer method allows a longer transfer distance of the transposition flap. In our practice, the advancement distance can achieve 6–10 mm. The transverse width of the flap was abandoned and the longitudinal length of the flap was obtained. The defects were evenly distributed on each side of the parallelogram to achieve sufficient transfer distance to cover the exposed bone and tissues.

The repair of fingertip defects with artery island flaps is a relatively simple and safe operation [10]. Homodigital island flaps are also pedicled with the finger artery. We harvested the flap from the palmar side of the finger and pushed the flap forward to cover the wound. The flap includes a lateral proper digital artery and a digital nerve [17]. The parallelogram flaps do not need to require stripping the artery, After a careful preoperative design of the parallelogram flap, we abandon the finger’s width and retain the length, successfully achieving the purpose of the operation and reduce the operation time. In our study, the operation time was obviously shorter in the Group A than in the Group B.

This article provided a detailed description of a modified flap for the surgical management of fingertip defects. The transfer flap was incised closely to the bone surface of the distal phalanx, and the interphalangeal artery was not damaged during stripping [18, 19], which is key to flap survival. Venous outflow is maintained by venules and capillaries in the perivascular adipose tissues through a retrograde fashion [20]. Therefore, if the interphalangeal artery is well protected during the flap design, the flap survival can be assured more confidently, as evidenced in our analyses that all our parallelogram flaps had survived postoperatively.

The reconstructive surgery for fingertip injury aims to obtain stable tissue coverage, achieve acceptable appearance, restore sensitivity, maintain finger length and resume normal physical activity promptly [21]. Some patients with homodigital island flaps had very obvious donor site scarring and skin sinking with poor aesthetics, and they were not very satisfied with the appearance of their fingers [22]. The incision of the parallelogram flap is distributed at both sides of the fingertip, and therefore the scar is at the sides of the finger. The patients with parallelogram flaps did not complain about the appearance of the fingers, and the MHQ (appearance) scores were significantly different between the groups.

The practice of sensory or non-sensory reconstruction of fingers remains controversial and debatable among hand surgeons. Studies have reported an average of 10 mm in the static two-point discrimination test when a “senseless” reverse digital artery island flap has been performed [23, 24]. Conversely, other studies have demonstrated a normal static two-point discrimination test (1–5 mm) following neurovascular island flaps [25, 26]. The findings of these studies indicate a reduced ability of flaps to restore sensation in the absence of nerve connections [27–29]. In parallelogram flaps and homodigital island flaps, the digital nerve can usually be preserved, so two types of operative method both provided a good sensory reconstruction of fingers, leading to satisfactory recovery in the finger movement, strength, etc.

## Conclusions

The reconstruction using parallelogram flaps is a easier and more versatile treatment with better functions, less morbidity and better aesthetics. This method is a better choice for reconstruction of fingertip injury.

## Data Availability

The datasets of the current study are available from the corresponding author upon reasonable request.

## Abbreviations

2PD: Two-point discrimination
TAM: Total active movement
MHQ: Michigan hand questionnaire
